# Nutraceuticals and phytoceuticals in the treatment of schizophrenia: a systematic review and network meta-analysis

**DOI:** 10.21203/rs.3.rs-3787917/v1

**Published:** 2024-01-12

**Authors:** Michele Fornaro, Claudio Caiazza, Martina Billeci, Michael Berk, Wolfgang Marx, Vicent Balanzá-Martínez, Michele De Prisco, Rosanna Pezone, Giuseppe De Simone, Niccolo’ Solini, Felice iasevoli, Fabrice Berna, Guillaume FOND, Laurent Boyer, Andre F Carvalho, Elena Dragioti, Jess Fiedorowicz, Andrea de Bartolomeis, Christoph Correll, Marco Solmi

**Affiliations:** Federico II University of Naples; University of Naples Federico II; University of Naples Federico II; Deakin University, Australia; Deakin University; University of Valencia; University of Naples Federico II; University of Naples Federico II; University of Naples Federico II; APHM; APHM; APHM; APHM; Innovation in Mental and Physical Health and Clinical Treatment (IMPACT) Strategic Research Centre, School of Medicine, Barwon Health, Deakin University, Geelong, VIC, Australia; Innovation in Mental and Physical Health and Clinical Treatment (IMPACT) Strategic Research Centre, School of Medicine, Barwon Health, Deakin University, Geelong, VIC, Australia; Donald and Barbara Zucker School of Medicine at Hofstra/Northwell; Donald and Barbara Zucker School of Medicine at Hofstra/Northwell; Donald and Barbara Zucker School of Medicine at Hofstra/Northwell; Donald and Barbara Zucker School of Medicine at Hofstra/Northwell; uOttawa

**Keywords:** Nutraceuticals, phytoceuticals, schizophrenia, treatment-resistance, systematic review, nutritional psychiatry, network meta-analysis, psychiatry, psychosis

## Abstract

**Background:**

Sub-optimal response in schizophrenia is frequent, warranting augmentation strategies over treatment-as-usual (TAU).

**Methods:**

We assessed nutraceuticals/phytoceutical augmentation strategies via network meta-analysis. Randomized controlled trials in schizophrenia/schizoaffective disorder were identified via the following databases: PubMed, MEDLINE, EMBASE, Scopus, PsycINFO, CENTRAL, and ClinicalTrials.gov. Change (Standardized Mean Difference=SMD) in total symptomatology and acceptability (Risk Ratio=RR) were co-primary outcomes. Secondary outcomes were positive, negative, cognitive, and depressive symptom changes, general psychopathology, tolerability, and response rates. We conducted subset analyses by disease phase and sensitivity analyses by risk of bias and assessed global/local inconsistency, publication bias, risk of bias, and confidence in the evidence.

**Results:**

The systematic review included 49 records documenting 50 studies (n=2,384) documenting 22 interventions. Citicoline (SMD=−1.05,95%CI=−1.85; −.24), L-lysine (SMD=−1.04,95%CI=−1.84;−.25), N-acetylcysteine (SMD=−.87,95%CI=−1.27;−.47) and sarcosine (SMD=−.5,95%CI=−.87−.13) outperformed placebo for total symptomatology. High heterogeneity (tau^2^=.10, I^2^=55.9%) and global inconsistency (Q=40.79, df=18, p=.002) emerged without publication bias (Egger’s test, p=.42). Sarcosine improved negative symptoms (SMD=−.65, 95%CI=−1.10; −.19). N-acetylcysteine improved negative symptoms (SMD=−.90, 95%CI=−1.42; −.39)/general psychopathology (SMD=−.76, 95%CI=−1.39; −.13). No compound improved total symptomatology within acute phase studies (k=7, n=422). Sarcosine (SMD=−1.26,95%CI=−1.91; −.60), citicoline (SMD=−1.05,95%CI=−1.65;−.44), and N-acetylcysteine (SMD=−.55,95%CI=−.92,−.19) outperformed placebo augmentation in clinically stable participants. Sensitivity analyses removing high-risk-of-bias studies confirmed overall findings in all phases and clinically stable samples. In contrast, the acute phase analysis restricted to low risk-of-bias studies showed a superior effect vs. placebo for N-acetylcysteine (SMD=−1.10,95%CI=−1.75,−.45), L-lysine (SMD=−1.05,95%CI=−1.55,−.19), omega-3 fatty acids (SMD=−.83,95%CI=−1.31,−.34) and withania somnifera (SMD=−.71,95%CI=−1.21,−.22). Citicoline (SMD=−1.05,95%CI=−1.86,−.23), L-lysine (SMD=−1.04,95%CI=−1.84,−.24), N-acetylcysteine (SMD=−.89,95%CI=−1.35,−.43) and sarcosine (SMD=−.61,95%CI=−1.02,−.21) outperformed placebo augmentation of TAU (“any phase”). Drop-out due to any cause or adverse events did not differ between nutraceutical/phytoceutical vs. placebo+TAU.

**Conclusions:**

Sarcosine, citicoline, and N-acetylcysteine are promising augmentation interventions in stable patients with schizophrenia, yet the quality of evidence is low to very low. Further high-quality trials in acute phases/specific outcomes/difficult-to-treat schizophrenia are warranted.

## Introduction

Schizophrenia and related disorders affect 23,6 million people worldwide, accounting for a significant burden ^1, 2^. People with schizophrenia often require long-term treatment with complex pharmacotherapy ^3^, especially in treatment-resistant cases ^4^. Nevertheless, up to 40% of patients fail to respond to standard antipsychotic treatment ^5^. While significant research is ongoing, currently, no novel mechanism-of-action medications for schizophrenia have been approved besides postsynaptic dopamine receptor antagonism ^6^. Hence, even small additional benefits from safe augmentation strategies are of potential value ^7^. Although 42 different pharmacologic augmentation strategies of antipsychotic agents were assessed in a previous umbrella review, the results were inconclusive due to the limited quality of the meta-analyzed studies and significantly greater effect sizes in lower-quality studies ^8^.

Nutraceuticals and phytoceuticals are increasingly used among non-pharmacological treatments due to their favorable safety and tolerability ^9, 10^. Yet, conclusive results are needed to establish their potential role in treating schizophrenia ^11^. Historically, the term nutraceutical has had various definitions ^12, 13^. Per previous umbrella reviews in this field, we refer to any nutrient-based intervention, such as vitamins, minerals, amino acids, and fatty acids ^14^. We also define phytoceuticals as any intervention that uses plant-based compounds such as herbal formulations ^15^.

Existing meta-analytic evidence ^14, 15^ and the most current World Federation of Societies of Biological Psychiatry (WFSBP) and Canadian Network for Mood and Anxiety Treatments (CANMAT) clinical guidelines ^11^ inform about the efficacy, safety, and tolerability of different nutraceuticals and phytoceuticals in the treatment of schizophrenia. However, no network meta-analysis (NMA) has been conducted to assess these compounds’ efficacy, acceptability, and tolerability in schizophrenia directly, except for a recent Bayesian NMA appraising selected compounds ^16^.

Therefore, the present NMA aimed to appraise the efficacy, acceptability, and tolerability of multiple nutraceuticals and phytoceuticals, either as mono- or augmentation therapy, in treating schizophrenia.

## Methods

The present systematic review (SR) and NMA evaluated nutraceutical and phytoceutical interventions in schizophrenia using randomized controlled trials (RCTs). The study followed the Preferred Reporting Items for Systematic Reviews and Meta-Analyses extension statement for network meta-analysis (PRISMA-NMA) ^17^. The protocol was registered via PROSPERO (CRD42022375946).

### Search strategy and selection criteria

We systematically searched PubMed/MEDLINE, EMBASE, Scopus, PsycINFO, Cochrane CENTRAL, and ClinicalTrials.gov from journal inception to October 17th, 2023. No language restriction was applied. The following search terms were adopted and augmented across different databases: psychosis, schizo*, nutraceuticals, phytoceuticals, and randomized controlled trials. The appendix (S1) details the search strategy. The electronic search was supplemented by a manual search of the reference list of all retrieved trials and relevant SRs and meta-analyses to identify additional relevant RCTs. Two authors (MB and MF) independently screened the papers and extracted data. Any disagreement was resolved by consensus with a third author (CC).

Inclusion criteria were: i) RCT; ii) inclusion of any nutraceutical/phytoceutical treatment, either as monotherapy or augmentation of treatment as usual (TAU), compared to either another nutraceutical/phytoceutical compound, placebo, TAU, or placebo + TAU; iii) involving adult (age ≥ 18 years) in- or outpatients with a primary diagnosis of schizophrenia or schizoaffective disorder, according to the Diagnostic and Statistical Manual of Mental Disorders (DSM), any edition/text revision, or International Classification of Disease (ICD), any edition; iv) providing quantitative data about the change in disease-specific symptomatology, measured through adequate rating scales, treatment response, all-cause drop-outs (acceptability) and, intolerability-related drop-outs (tolerability).

Excluded were studies with i) observational or non-randomized design; ii) without a suitable control group (e.g., studies comparing two different doses of the same nutraceutical/phytoceutical molecule); iii) involving < 10 patients per arm, consistent with most WFSBP/CANMAT guidelines ^11^; iv) crossover design not providing pre-cross-over data (otherwise included in the SR portion only); v) special populations, like first-episode psychosis (since a definitive diagnosis of schizophrenia is unclear), high-/ultra-high risk populations, pediatric populations (age < 18 years); vi) unstratified results for different diagnostic populations (e.g., schizophrenia and bipolar disorder merged altogether).

### Study selection, extraction, and outcomes

The following data were extracted: author(s), year, nutraceutical/phytoceutical and control interventions, mean dose of the active intervention, study design (parallel-group or crossover), trial duration, sample size (total, cases, controls), mean age and % of females in each arm, diagnostic criteria and (semi)structured interview, acceptability, tolerability, efficacy measures as reported in RCTs for the primary outcome (mean difference in scoring at endpoint from baseline, response rate). We also recorded whether the outcomes were reported per intention-to-treat (ITT) (i.e., last observation carried forward [LOCF]) or per-protocol (i.e., completers). All outcomes were measured at the study endpoint. For crossover RCTs, only the pre-cross-over effect was considered. The following were deemed co-primary outcomes: i) efficacy, as the mean change in the total score of schizophrenia-specific rating scales, and ii) acceptability. Secondary outcomes were: i) mean change in positive, negative, cognitive, and depressive symptoms and general psychopathology, ii) intolerability-related drop-out, and iii) treatment response. Contact with study authors was attempted when data were unavailable.

### Risk of bias, confidence in the evidence, and quality appraisal

The risk of bias was assessed using the Cochrane Risk of Bias tool, second edition (RoB2) ^18^. Confidence in the evidence for the primary outcomes was evaluated within the Confidence In Network Meta-Analysis (CINeMA) framework ^19^. Finally, an adapted version of the AMSTAR plus content total score ^8^ was employed to rate the quality of the included RCTs, weighted for the number of comparisons (score range = 0–7, owing to the following adapted items: 1) Blindness yes/no (score range 0–1); 2) sample size (n < 20 score = 0, n = 20–100 score 1, n > 100 score = 2); 3) findings replicated in at least one comparison (1 point) and n > 20 in at least one study arm (1 point); 4) ITT (no = 0, yes = 1); 5) High (score = 0) or low risk (score = 1) of bias at the Rob2 regressed against the primary efficacy outcome effect size (change in total PANSS score: SMD, 95%CI).

### Data analysis

We performed a random-effect NMA within the frequentist framework using the netmeta package v.2.1–0, RStudio version 4.2.1. We computed standardized mean difference (SMD) for continuous outcomes and risk ratio (RR) for binary ones. For studies failing to report the mean changes but only baseline and endpoint data, the SMD and standard deviation (SD) were estimated for each study arm using the methods outlined in the Cochrane Handbook ^20^.

The homogeneity of direct evidence (within each pairwise comparison) was assessed using tau^2^. Global inconsistency was evaluated by considering a full-design-by-treatment model. Local inconsistency was measured with a node-splitting approach to assess the agreement between direct and indirect estimates for each outcome. The study node was the nutraceutical/phytoceutical molecule. Publication bias was measured for each outcome by visually examining the funnel plot and Egger’s test for studies with ≥ 10 participants ^21^.

The relative ranking of different nutraceutical/phytoceutical interventions was estimated using the surface under the cumulative ranking curves (SUCRA). The higher the SUCRA value, the better the rank of the intervention within the accounted series of interventions.

The following subgroup analyses were planned for efficacy outcomes: acute vs. maintenance phase schizophrenia and clozapine vs. non-clozapine antipsychotics. The following sensitivity analyses were planned for the co-primary outcomes: i) excluding studies with a high risk of bias and ii) focusing on schizophrenia studies only.

## Results

**Figure 1:**
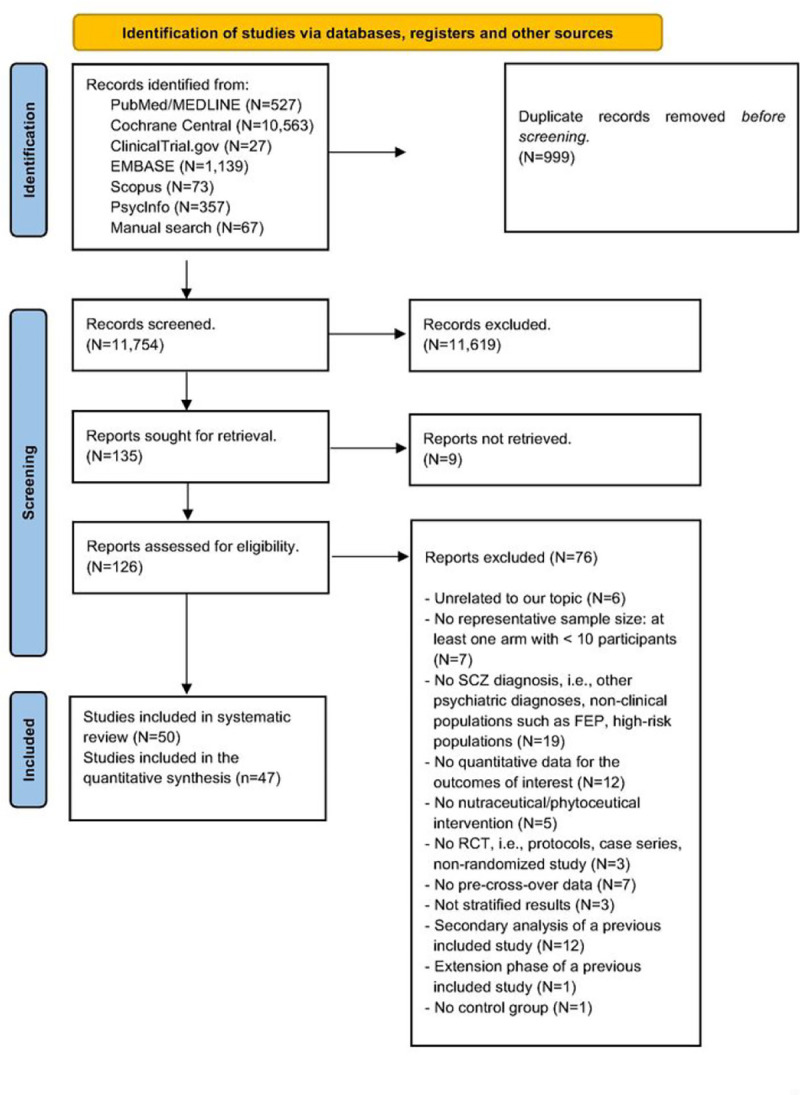
PRISMA 2020 flow diagram.

The systematic search yielded 12,744 results, reduced to 11,754 after 990 duplicates were automatically detected and removed. Title and abstract screening led to 135 records eligible for the full-text screening. After the full-text assessment, 50 records were included in the SR ^22–70^. One record provided two independent studies ^34^. An exhaustive list of excluded studies with reasons is reported in the appendix, S8. The PRISMA flow diagram is summarized in Fig. 1. All included studies compared nutraceutical/phytoceutical compounds + TAU to placebo + TAU. Three studies did not enter the NMA synthesis: two^37, 39^ due to the lack of TAU and one^22^ due to unreliable TAU (Table 1).

All included studies were parallel-group RCTs, except two crossover design studies ^22, 68^. The median number of participants was n = 60 (interquartile range, n = 40), range, n = 20–157). Most studies ranged from four to 24 weeks, except for three that lasted 26 weeks ^36^, 52 weeks ^32^, and two years ^39^, respectively. The latter ^39^ was a maintenance study involving patients who discontinued the antipsychotic treatment, undergoing randomization to omega-3 + α-lipoic acid or placebo, thus not being appropriate for the quantitative synthesis.

Most of the included studies were conducted in Asia (29 studies), followed by North America (12 studies), Europe (five studies), Africa (two studies), and Australia (two studies).

In all included studies, the efficacy of nutraceuticals or phytoceutical compounds was assessed through the score on the Positive and Negative Syndrome Scale (PANSS) ^71^, Scale for the Assessment of Positive Symptoms (SAPS) ^72^, Scale for the Assessment of Negative Symptoms (SANS) ^73^, and the Clinical Global Impression-Improvement module (CGI-I) ^74^, different cognitive batteries, and functioning scales.

Most studies were conducted on patients with chronic schizophrenia, with baseline PANSS total scores ranging from 48 ^60^ to 114 ^31^. The phase of illness and the setting of the study have been detailed in Table 1. Finally, the lack of corresponding data precluded a subgroup analysis for clozapine studies.

**Figure 2:**
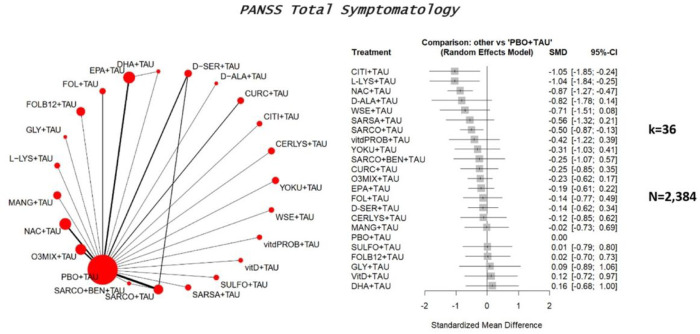
Forest plot showing effects of nutraceuticals and phytoceuticals on change in schizophrenia-specific **total** symptomatology, compared to placebo+TAU. CERLYS=Cerebrolysin; CITI=Citicoline; CURC=Curcumin; D-ALA=D-Alanine; DHA=Docosahexaenoic acid; D-SER=D-Serine; EPA=Eicosapentaenoic acid; FOL=Folate; FOLB12=Folates+Vitamin B12; GLY=Glycine; L-LYS=L-Lysine; MANG=Mangosteen; N-ACETYLCYSTEINE=N-Acetylcysteine; O3MIX=Omega-3; PBO=Placebo; WSE=Whitania Somnifera Extract; SARCO=Sarcosine; SARCO+BEN=Sarcosine+Sodium Benzoate; SARSA=Sarsasapogenin; SULFO=Sulforaphane; VitD=Vitamin D; vitdPROB=Vitamin D+Probiotics; YOKU=Yokukansan. Note: data refers to any of the disease.

**Figure 3:**
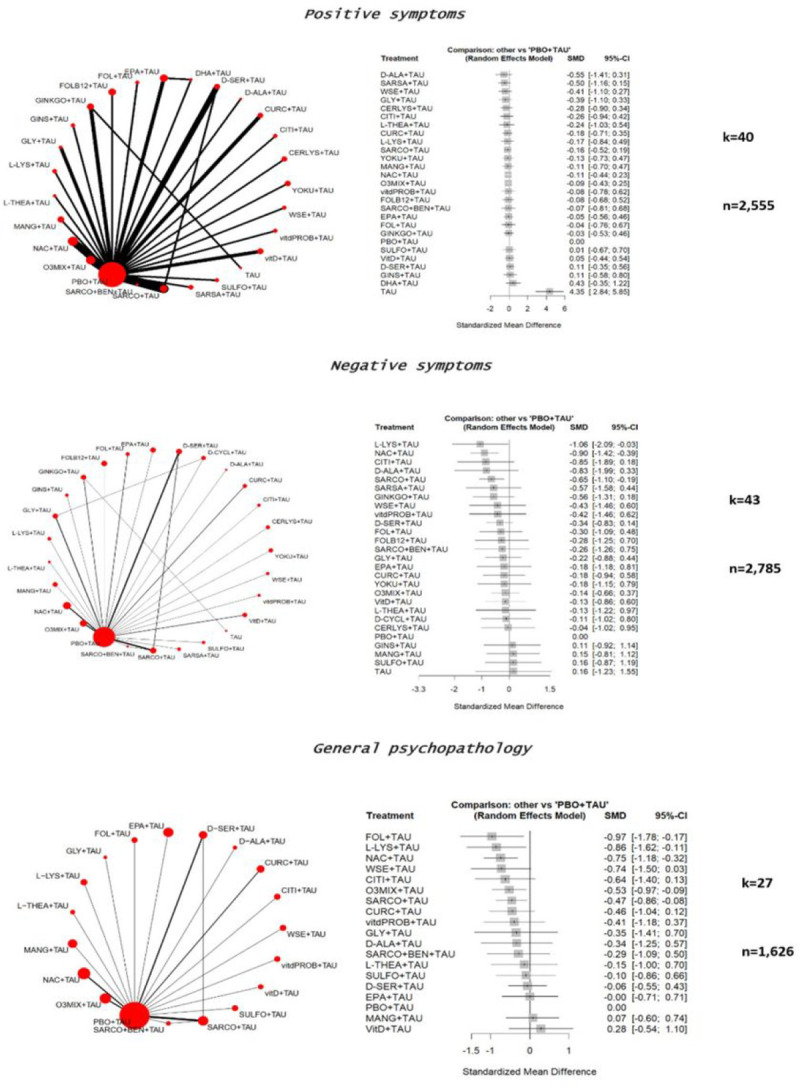
Forest plot showing effects of nutraceuticals and phytoceuticals on change in **positive**and **negative** symptoms and symptomatology. CERLYS=Cerebrolysin; CITI=Citicoline; CURC=Curcumin; D-ALA=D-Alanine; DHA=Docosahexaenoic acid; D-SER=D-Serine; EPA=Eicosapentaenoic acid; FOL=Folate; FOLB12=Folates+Vitamin B12; GINKGO=Ginkgo Biloba; GINS=Ginseng extract; GLY=Glycine; L-LYS=L-Lysine; L-THEA=L-Theanine; MANG=Mangosteen; N-ACETYLCYSTEINE=N-Acetylcysteine; O3MIX=Omega-3; PBO=Placebo; WSE=Whitania Somnifera Extract; SARCO=Sarcosine; SARCO+BEN=Sarcosine+Sodium Benzoate; SARSA=Sarsasapogenin; SULFO=Sulforaphane; VitD=Vitamin D; vitdPROB=Vitamin D+Probiotics; YOKU=Yokukansan. Note: data refers to any phase of the disease.

**Figure 4:**
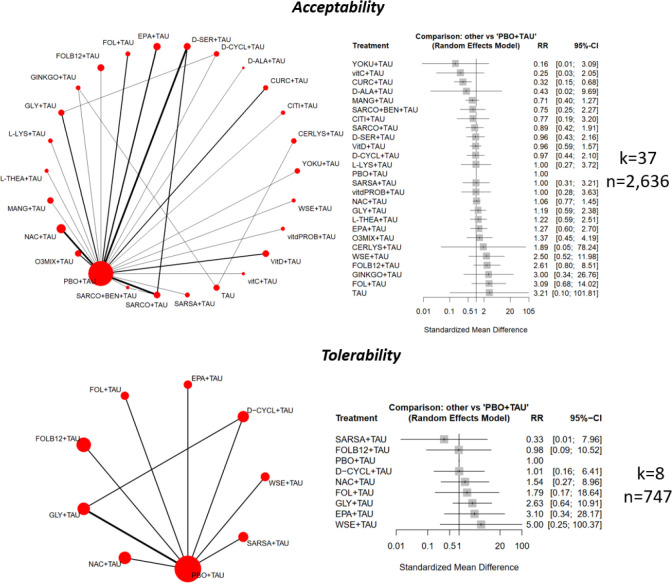
Forest plot showing effects of nutraceuticals and phytoceuticals on acceptability and tolerability. CERLYS=Cerebrolysin; CITI=Citicoline; CURC=Curcumin; D-ALA=D-Alanine; DHA=Docosahexaenoic acid; D-SER=D-Serine; EPA=Eicosapentaenoic acid; FOL=Folate; FOLB12=Folates+Vitamin B12; GINKGO=Ginkgo Biloba; GINS=Ginseng extract; GLY=Glycine; L-LYS=L-Lysine; L-THEA=L-Theanine; MANG=Mangosteen; N-ACETYLCYSTEINE=N-Acetylcysteine; O3MIX=Omega-3; PBO=Placebo; WSE=Whitania Somnifera Extract; SARCO=Sarcosine; SARCO+BEN=Sarcosine+Sodium Benzoate; SARSA=Sarsasapogenin; SULFO=Sulforaphane; VitD=Vitamin D; vitdPROB=Vitamin D+Probiotics; YOKU=Yokukansan. Note: data refers to any phase of the disease.

A qualitative synthesis of the results of all included RCTs is reported in Table 1. The main results are summarized in Figs. 2–4.

Regarding the risk of bias, 11 studies (22% of the records) were rated as having some concerns, 27 studies as low-risk (54%), and 12 as high risk of bias (24%) (e-Table 1).

### Network meta-analyses of nutraceuticals and phytoceuticals as an augmentation to treatment as usual

#### PANSS total symptomatology

Thirty-six studies with 22 treatments encompassing 2,384 patients were included in the NMA for change in total symptomatology. Specifically, the following augmentations of TAU relied on one RCT only: cerulysine, citicoline, D-alanine, DHA, folate + Vitamin B12, glycine, L-lysine, mangosteen, sarcosine + sodium benzoate, sarsapogenein, sulforaphane, vitamin D, vitamin D + probiotics, whitania somnifera, and yokukansan. The following augmentations of TAU relied on two RCTs: curcumin and folate. The following augmentations of TAU relied on three RCTs: D-serine. The following augmentations of TAU relied on four RCTs: NAC, EPA, and omega-3. Sarcosine relied on six RCTs.

As an adjunct to TAU, most nutraceutical or phytoceutical compounds failed to outperform placebo + TAU regarding total symptomatology; see the forest plot (Fig. 2 and the league Table; e-Table 2). A statistically significant difference was found only for the following supplements: citicoline (SMD=−1.05, 95%CI=−1.85; − .24), L-lysine (SMD=−1.04, 95%CI=−1.84; − .25); N-acetylcysteine (SMD=−.87, 95%CI=−1.27; − .47), sarcosine (SMD=−.5, 95%CI=−.87; − .13) (e-Figure 1). Citicoline outperformed DHA, L-lysine outperformed Vit-D and DHA, N-acetylcysteine outperformed omega-3, EPA, D-serine, mangosteen, folate + Vit.B12, Vitamin D and DHA (e-Table 2). Considerable heterogeneity was present (tau^2^ = .10, I^2^ = 55.9%) and global inconsistency (Q = 40.79, df = 18, p = .002). No publication bias was detected (Egger’s test, p = .42) (e-Figure 2).

Considering acute phase studies (k = 7, n = 422), all evaluated compounds (L-Lysine, N-acetylcysteine, withania somnifera, sarcosine, omega-3, Vitamin D, and D-serine) failed to show a significant effect on the total symptomatology, when compared to placebo augmentation (e-Figure 3 & e-Table 3; Section S5). Considering clinically stable patients (k = 12, n = 850), a significant difference was found for the following augmentation strategies vs. placebo: sarcosine (SMD=−1.26, 95%CI=−1.91; − .60), citicoline (SMD=−1.05, 95%CI=−1.65, − .44), and N-acetylcysteine (SMD=−.55, 95%CI=−.92, − .19) (e-Figure 4). Also, regarding head-to-head comparisons, sarcosine outperformed all other interventions except citicoline and N-acetylcysteine; citicoline outperformed all other active compounds except sarcosine, N-acetylcysteine, and yokukansan (e-Table 4).

No significant publication bias was found (p = .54) (e-Figure 5). Such findings were consistently supported by sensitivity analyses removing high risk-of-bias studies in all phase analyses (k = 26, n = 1,716) (e-Figure 6, e-Table 5). In contrast, the acute phase analysis without high-risk-of-bias studies (k = 6, n = 355) showed a significant effect for the following augmentations vs. placebo: N-acetylcysteine (SMD=−1.10, 95%CI=−1.75, − .45), L-lysine (SMD=−1.05, 95%CI=−1.55, − .19), omega-3 (SMD=−.83, 95%CI=−1.31; − .34) and withania somnifera (SMD=−.71, 95%CI=−1.21, − .22) (e-Figure 7 & e-Table 6). Sensitivity analysis of clinically stable patients (k = 9, n = 667) was concordant with the main analysis of studies other than high risk-of-bias (e-Figure 8 & e-Table 7).

Considering people with schizophrenia in all phases of the disease (k = 26, n = 1,542), citicoline (SMD=−1.05, 95%CI=−1.86, − .23), L-lysine (SMD=−1.04, 95%CI=−1.84, − .24), N-acetylcysteine (SMD=−.89, 95%CI=−1.35, − .43) and sarcosine (SMD=−.61, 95%CI=−1.02, − .21) outperformed placebo augmentation of TAU (e-Figure 9 & e-Table 8). In analysis of acute phase schizophrenia analysis (k = 5, n = 285), N-acetylcysteine (SMD=−1.10, 95%CI=−1.75, − .45) and L-Lysine (SMD=−1.05, 95%CI=−1.55, − .56) outperformed placebo (e-Figure 10 & e-Table 9). Considering clinically stable patients with schizophrenia (k = 8, n = 582), TAU augmentation with either sarcosine (SMD=−1.26, 95%CI=−1.83, − .68), citicoline (SMD=−1.05, 95%CI=−1.56, − .53), and N-acetylcysteine (SMD=−.41, 95%CI=−.75, − .08) outperformed placebo + TAU, supporting the main analysis findings (e-Figure 11 & e-Table 10).

#### Positive symptoms

Regarding positive symptoms (measured through PANSS positive subscale or SAPS), 40 studies with 27 treatments encompassing 2,555 patients were included in the main analysis, encompassing all phases of the disease. Specifically, the following augmentations of TAU relied on one RCT only: cerulysine, citicoline, D-alanine, DHA, folate, folate + Vitamin B12, ginseng, L-lysine, L-theanine, mangosteen, sarcosine + sodium benzoate, sarsapogenein, sulforaphane, vitamin D + probiotics, whitania somnifera, and yokukansan. The following augmentations of TAU relied on two RCTs: curcumin and glycine. The following augmentations of TAU relied on three RCTs: D-serine and ginkgo biloba. The following augmentations of TAU relied on four RCTs: NAC and omega-3. Sarcosine relied on five RCTs.

Along with placebo augmentation, every compound outperformed TAU monotherapy. However, no compound outperformed placebo + TAU (e-Table 11). Moderate heterogeneity was present (tau^2^ = .06, I^2^ = 42.9%), while significant global inconsistency was found (Q = 29.77, df = 17, p = .0281). No publication bias was detected (Egger’s test, p = .85) (e-Figure 13). Considering acute phase and clinically stable studies, all compounds overlapped placebo (e-Figure 14–15 & e-Table 12–13).

#### Negative symptoms

The following augmentations of TAU relied on one RCT only: cerulysine, citicoline, D-alanine, D-cycloserine, EPA, folate + Vitamin B12, ginseng, L-lysine, L-theanine, mangosteen, sarcosine + sodium benzoate, sarsapogenein, sulforaphane, vitamin D + probiotics, whitania somnifera, and yokukansan. The following augmentations of TAU relied on two RCTs: curcumin and vitamin D + folate. The following augmentations of TAU relied on three RCTs: glycine. The following augmentations of TAU relied on four RCTs: NAC and omega-3. D-serine and sarcosine relied on five and six RCTs, respectively.

Regarding negative symptoms (measured through PANSS negative subscale or SANS), considering all phases of disease (k = 43, n = 2,785), among 25 treatments, a significant effect was found for the following augmentation strategies, compared to placebo: L-Lysine (SMD=−1.06, 95%CI=−2.09; − .03), N-acetylcysteine (SMD=−.90, 95%CI=−1.42; − .39), and sarcosine (SMD=−.65, 95%CI=−1.10; − .19). All other compounds did not outperform the placebo augmentation (e-Figure 16 & e-Table-14).

High heterogeneity was found (tau^2^ = .213, I^2^ = 70.8%). Global inconsistency was high (Q = 71.89, df = 21, p < .001). No publication bias was detected (Egger’s test, p = .09) (e-Figure 17).

Regarding acute phase studies (k = 7, n = 422), only N-acetylcysteine (SMD=−1.18, 95%CI=−1.83; − .52) and L-Lysine (SMD=−1.06, 95%CI=−1.55; − .56) outperformed placebo augmentation (e-Figure 18). N-acetylcysteine and L-Lysine outperformed sarcosine, Omega-3 mixture, and Vitamin D (e-Table 15).

In samples of clinically stable patients (k = 18, n = 1,190), only sarcosine (SMD=−2.30, 95%CI=−3.26; −1.34) and citicoline (SMD=−.85, 95%CI=−1.70; − .01) outperformed placebo. N-acetylcysteine overlapped with the placebo effect, while L-lysine did not enter the pertinent analysis (e-Figure 19). Sarcosine also outperformed all other compounds (e-Table 16). No publication bias was detected (Egger’s test, p = .08) (e-Figure 20).

#### General Psychopathology

Regarding PANSS general psychopathology (k = 27, n = 1,626), among 18 augmentation strategies, Folate (SMD=−.97, 95%CI=−1.78; − .17), L-Lysine (SMD=−.86, 95%CI=−1.62; − .11), and N-acetylcysteine (SMD=−.75, 95%CI=−1.18; − .32) outperformed placebo, while all other compound overlapped its effects (e-Figure 21 & e-Table 17). The following augmentations of TAU relied on one RCT only: citicoline, D-alanine, EPA, folate, glycine, L-lysine, L-theanine, mangosteen, sarcosine + sodium benzoate, sulforaphane, vitamin D, and whitania somnifera. Curcumin relied on two RCTs, while D-serine, NAC, and omega-3 relied on three. Sarcosine relied on five RCTs.

Moderate heterogeneity emerged (tau^2^ = .088, I^2^ = 51%), as well as significant global inconsistency (Q = 22.46, df = 11, p = .02). No publication bias was detected (Egger’s test, p = .17) (e-Figure 22).

Regarding acute phase studies (k = 6, n = 355), omega-3 (SMD=−.91, 95%CI=−1.39; − .42), L-lysine (SMD=−.86, 95%CI=−1.34; − .38), sarcosine (SMD=−.78, 95%CI=−1.43; − .12), N-acetylcysteine (SMD=−.76, 95%CI=−1.39; − .13) and withania somnifera (SMD=−.74, 95%CI=−1.24; − .24) outperformed placebo (e-Figure 23) and Vitamin D (e-Table 18). Regarding clinically stable patients’ analysis (k = 10, n = 563), sarcosine (SMD=−.97, 95%CI=−1.53; − .42), citicoline (SMD=−.64, 95%CI=−1.13; − .14), curcumin (SMD=−.47, 95%CI=−.87; − .06), and N-acetylcysteine (SMD=−.36, 95%CI=−.70; − .03) outperformed placebo (e-Figure 24 & e-Table 19). Low heterogeneity (tau^2^ = 0, I^2^ = 0%) and significant global inconsistency (Q = .37, df = 2, p = .83) emerged.

#### Depressive and cognitive symptoms

Regarding depressive symptoms (k = 4, n = 274), all assessed augmentation strategies (N-acetylcysteine, mangosteen, D-alanine, and ginseng) failed to differ from placebo (e-Figure 25 & e-Table 20). The following compounds relied upon one RCT only: D-alanine, ginseng, mangosteen, and NAC.

Regarding cognitive symptoms (k = 7, n = 344), N-acetylcysteine (SMD=−1.02, 95%CI=−1.56; − .49) augmentation outperformed placebo (e-Figure 26). N-acetylcysteine outperformed yokukansan, sarcosine, and D-serine (e-Table 21). Similar findings emerged in evaluating clinically stable patients (k = 3, n = 197) (e-Figure 27 & e-Table 22). The following augmentations of TAU relied on one RCT only: D-alanine, NAC, and yokukansan. D-serine relied on two RCTs, while sarcosine relied on three.

#### Clinical Response Rate

Regarding response rates (as per author’s definition) (k = 4, n = 274), only withania somnifera (RR = 3.00, 95%CI = 1.77; 5.09) and curcumin (RR = 9.50, 95%CI = 2.43; 37.13) augmentations outperformed placebo, while eicosatetraenoic Acid (EPA), docosahexaenoic acid (DHA), and Omega-3 failed to differ from placebo (e-Figure 28 & e-Table 23). Specifically, the appraised compounds relied on only one RCT: curcumin, DHA, EPA, omega-3, and whitania somnifera.

#### Acceptability and Tolerability Analysis

Regarding acceptability, 37 studies were included, accounting for 2,636 patients. None of the 24 nutraceutical or phytoceutical augmentation strategies significantly differed from the placebo regarding the RR of drop-out due to any cause (e-Figure 29 & e-Table 24). No heterogeneity was found (tau^2^ = 0, I^2^ = 0%). Global inconsistency was not statistically significant (Q = 9.33, df = 15, p = .86). No publication bias was detected (Egger’s test, p = .90) (e-Figure 30). Similar findings emerged from the sensitivity analyses without high risk-of-bias studies (e-Figure 31 & e-Table 25) and with people with schizophrenia only (e-Figure 32 & e-Table 26).

Concerning tolerability (k = 8, n = 747), none of the eight assessed augmentation strategies differed from the placebo regarding the RR of drop-out due to adverse events (e-Figure 33 & e-Table 27). Finally, there was insufficient information to compute the inefficacy (discontinuation due to lack of efficacy), set as a secondary outcome.

#### Confidence in evidence

The CINeMA confidence in evidence and its methods is summarized in Appendix S6. Regarding both PANSS total symptom and acceptability analyses, confidence in evidence was low/very low throughout all the comparisons (e-Tables 29–30). Likewise, meta-regression analysis consistently documented a trend for lower SMDs as the adapted AMSTAR content score increased across different outcomes (e.g., PANSS total symptoms change = β=−0.0245, 95%CI = .0.0866, −0.1943, p = .77, R^2^ = .34, N = 22).

## Discussion

This NMA concurrently appraised 22 nutraceutical and phytoceutical interventions for adjunctive treatment of schizophrenia and related conditions, expanding the series of compounds already appraised by previous work (although a handful of compounds could nonetheless not be included in the present report due to the stringent inclusion/exclusion criteria) ^14, 16^. Specifically, the present NMA also included N-Methyl-D-Aspartate receptor (NMDAR) modulators, such as D-alanine, D-serine, D-cycloserine, glycine, sarcosine (N-substituted glycine), and plant-derived compounds such as curcumin, in addition to vitamins (e.g. Folate, B12, D), N-acetylcysteine, and omega-3 fatty acids, otherwise included in the WFSBP and CANMAT clinical guidelines ^11^.

While the NMA showed that most of the appraised nutraceutical and phytoceutical compounds failed to provide a significant benefit compared to placebo augmentation of TAU, specific compounds showed promising beneficial effects as an antipsychotic-augmentation strategy, with favorable acceptability and tolerability profiles. N-acetylcysteine and sarcosine improved total and negative symptoms. PANSS general symptomatology improved with curcumin and N-acetylcysteine. Citicoline improved total symptomatology only.

While mechanistic understanding of these nutraceutical/phytoceutical interventions is ongoing, the efficacy of select agents seen in our NMA is concordant with the currently understood mechanisms of action of these interventions.

Specifically, translational evidence suggests a relevant role of the nitric oxide (NO) signaling pathway in schizophrenia pathogenesis, affecting glutamate and dopamine storage, uptake, and release, also increasing oxidative stress ^75^. In this regard, L-lysine competes with L-arginine for the cationic amino-acid transporter ^76^ on the blood-brain barrier, thus reducing the influx of L-arginine and, consequently, the overall arginine-based NO synthesis ^76^. Altered regulation of oxidative stress and neuroinflammatory mechanisms may be relevant to the pathogenesis and the progression of schizophrenia and related medical and psychiatric disorders, likely due to membrane and neuronal damage ^77, 78^. Moreover, a protracted inflammatory state activates indoleamine 2,3-dioxygenase, producing neuroactive metabolites that influence the dopaminergic and glutamatergic neurotransmission, inducing neurotoxic stimuli ^79, 80^. Furthermore, an imbalance in glutamate neurotransmission is increasingly relevant in pathogenic theories of schizophrenia ^81^. Such neurobiological pathways may explain why the appraised beneficial compounds (i.e., L-lysine, N-acetylcysteine, citicoline, sarcosine, and curcumin) appear to be efficacious in schizophrenia, considering that they share direct/indirect antioxidant, anti-inflammatory and glutamate modulatory activity.

Curcumin exerts antioxidant, neuroprotective, and anti-inflammatory properties ^82^, concordant with its beneficial effects on general symptoms. Citicoline is a precursor of acetylcholine and membrane phospholipids with neuroprotective, antioxidant, and anti-excitotoxic properties ^83^. Moreover, citicoline may exert anti-inflammatory activity via unclear mechanisms, likely reducing blood-brain barrier permeability ^84^.

N-acetylcysteine is a glutathione precursor with antioxidant and anti-inflammatory properties ^85^ and is already recommended for adjunctive treatment in schizophrenia (primarily for negative symptoms) at 1–3 g/day ^11^. N-acetylcysteine influences glutamate neurotransmission by enhancing NMDAR activity ^86^, and several studies showed that its supplementation improved the processing speed, attention, working memory, and executive functioning of patients with schizophrenia ^87–89^, consistent with results from a recent umbrella review of adjunctive therapies in schizophrenia ^90^.

Finally, sarcosine is a glycine transporter type-I inhibitor and NMDAR allosteric modulator, directly binding to the NMDAR glycine co-agonist site, enhancing NMDAR activity, and possessing neuroprotective properties ^91, 92^. However, previous studies (not included in our study due to our exclusion criteria) suggested that sarcosine may not be effective when used as an augmentation agent with clozapine. This lack of efficacy is likely due to the potential glycinergic and glutamatergic activity of clozapine itself^53, 93^, resulting in a “ceiling” effect when additional modulation of NMDA receptors is unlikely ^92^.

Hypothetically, a high-inflammatory/oxidative stress subset of people with schizophrenia may benefit from such supplementations. Since oxidation, inflammatory, and genetic biomarkers could guide the stratification of people with schizophrenia ^94, 95^, further nutraceutical/phytoceutical RCTs may adopt biomarkers as inclusion criteria to evaluate different efficacy profiles across various subsets ^90^.

## Limitations of the study

First, the main limitation of the present NMA is the low number of studies for each meta-analyzed agent. For example, for the primary efficacy outcome, for only five agents, more than one or two studies could be meta-analyzed, i.e., sarcosine: k = 5; and N-acetylcysteine, D-serine, EPA, and omega-3 (k = 4 each). Second, our findings rely on a limited number of comparisons, with most going via the placebo node, warranting future studies to draw firmer conclusions regarding the results. Third, high global inconsistency emerged across most comparisons, reflecting the trial duration heterogeneity and the underlying antipsychotic drugs and doses comprising TAU. Fourth, the limited number of studies also precluded feasible subgroup and sensitivity analyses to explain the observed inconsistency. Fifth, many studies lacked information regarding illness characteristics that could affect outcomes and inform subgroups, including illness phase (i.e., acute vs. clinically stable). Sixth, only 54% of the studies had a low risk of bias. Seventh, low and very low confidence in evidence emerged in the CINeMA, underscoring again that more high-quality studies are needed and that the results of this NMA need to be interpreted with caution, especially considering the trend for larger effect sizes documented by records with lower AMSTAR plus content score (adapted). Finally, caution in translating the results from this NMA to clinical care is also warranted because nutraceutical/phytoceuticals are often heterogeneous in composition, and the bioavailability and the effects of the same compound may vary across different preparations. No clear-cut guidance is often available because FDA and other regulatory agencies’ requirements for nutraceuticals and phytoceuticals differ from traditional drugs ^96^.

## Conclusions

Among many nutraceuticals tested as an augmentation treatment for schizophrenia, sarcosine (2gr/day), citicoline (2.5gr/day), and N-acetylcysteine (1.2–3.6gr/day) consistently show a beneficial effect for total and specific symptoms, almost exclusively in stable patients, with good overall acceptability. However, multiple limitations encourage caution in interpreting and translating the results into clinical practice. Larger and high-quality RCTs with a low risk of bias should investigate the effects of nutraceuticals in schizophrenia, especially using heterogeneous daily doses and formulations.

## Figures and Tables

**Table n.1:** Characteristics of included studies assessing nutraceuticals and phytoceuticals in treating schizophrenia.

Author(s), Year	Population, N=sample size, PANSS baseline, if provided	Mean age in years. Female %	Diagnosis DSM/ICD and type of interview	Duration and design; ITT/PP analysis	Sponsor	Nutraceutical/phytoceutical intervention
Atmaca M et al., 2005	Not better-specified phase of illness SCZ outpatients N=29	Age: 27.9 F: 78.9%	DSM IV semi-structured interview (unspecified)	Eight weeks, parallel. Intent-to-treat analysis	Not reported	Ginkgo biloba (300mg/day) +olanzapine (5 20mg/day; mean dose at week 8: 14.7mg/day)
Berk M. et al., 2008	Clinically stable SCZ outpatients N=140 45% on clozapine, 20% on olanzapine Mean PANSS baseline 64 and 64.4 for groups I and II, respectively	Age: 36.6 F: 30%	DSM IV-TR, MINI	24 weeks, parallel; Intent-to-treat analysis	A grant from the Stanley Medical Research Institute	N-acetylcysteine(2g/day) + antipsychotic treatment (mean CPZ-equivalent 716.4 mg/day)
Breier A. et al., 2018	Clinically stable SCZ Spectrum outpatients (SCZ, schizophreniform, schizoaffective, NOS) N=60 The mean PANSS baseline was 56.6 and 56.4 for groups I and II, respectively.	Age: 23.6 F: 21.66%	DSM IV-TR, SCID	52 weeks, parallel; intent-to-treat analysis	Funded by the Stanley Medical Research Institute (10T-002)	N-acetylcysteine (3.6g/day) +antipsychotics (mean CPZ-equivalent 130.9mg/day)
**Chen EYH et al., 2011**	Clinically stable SCZ outpatients N=64	Age: 43.41 F: 20.3%	DSM IV	4. weeks, parallel; intent-to-treat analysis	Supported by a research grant from Afexa Life Sciences	Ginseng(200mg/day) +antipsychotics (mean CPZ-equivalent 964.54 mg/day)
Hosseininasab Met al., 2021	Clinically stable SCZ outpatients with predominantly negative symptoms N=58 Mean baseline PANSS 66.8 and 70.6 for groups I and II, respectively	Age: 48.55 F: not reported	DSM-5, clinical interview based on DSM-5	16 weeks, parallel; per-protocol analysis	Grant number 83 from the Mazandaran University of Medical Sciences research council to N.H.	Nanocurcumin(160mg/day) +risperidone, perphenazine, or clozapine (mean CPZ-equivalent 950mg/day)
Miodownik C et al., 2019	Clinically stable SCZ outpatients with persistent negative symptoms (SANS>30) N=38 Mean baseline PANSS 99.8 and 91.5 for groups I and II, respectively	Age: 53.76 F: 34.2%	DSM-IV	24 weeks, parallel; intent-to-treat analysis	Not reported	Curcumin (3000mg/day) +antipsychotics (mean CPZ-equivalent 1009.1 mg/day)
**Roffman JL et al., 2017**	Not better-specified phase of illness SCZ outpatients N=55 Baseline PANSS 74.5 and 79.7 for groups I and II, respectively	Age: 45.54 F: 21.8%	DSM-IV-TR, SCID	12 weeks, parallel; intent-to-treat analysis	Funded by Pamlab and NIMH (R01MH101425, S10RR023043, S10RR023401, K24MH094614)	L-methyl-folate(15mg/day) +antipsychotics, antidepressants, or anticonvulsants
Roffman JL et al., 2013	Clinically stable SCZ outpatients with persistent symptoms N=140 PANSS > 60	Age: 45.5 F: 28.6%	DSM-IV-TR, SCID	16 weeks, parallel; intent-to-treat analysis	NIMH grant R01MH070831 and the Howard Hughes Medical Institute Early Career Physician-Scientist Award.	Folate (2mg/day) and vitamin B12 (400mcg/day) +antipsychotics, antidepressants, or anticonvulsants
* Levine et al., 2006	Clinically stable SCZ outpatients N=42 Mean baseline PANSS 94.4 and 93 in groups I and II, respectively	Age: 40 F: not reported	DSM-IV	12 weeks, crossover; per-protocol analysis	Stanley Medical Research Institute grant (to RHB, JL)	Folate (2mg/day), pyridoxine (25mg/day), and vitamin B12 f400mcg/day) + antipsychotics, lithium, anticonvulsants (mean CPZ-equivalent 900mg/day)
Hill M et al., 2011	Clinically stable SCZ outpatients with residual negative symptoms N=32 Mean baseline PANSS 69.6 and 75.3 for groups I and II, respectively	Age: 45.9 F: 18.7%	DSM-IV, clinical interview	12 weeks, parallel; per-protocol analysis	Funded by the NARSAD 2003 Investigator Award	Folate (2mg/day) +antipsychotics
Krivoy A et al., 2017	Acute SCZ outpatients treated with clozapine. N=47 Baseline PANSS >60	Age: 40.9 F: 31.9%	DSM-IV-TR	Eight weeks, parallel; intent-to-treat analysis	grant from Stanley Medical Research Institute (grant 08–13T)	Vitamin D (14000IU/week) +clozapine (414.6mg/day
Sheikmoonesi F et al., 2016	Not better-specified phase of illness SCZ outpatients with residual symptoms N=80	Age: 47.2 F: 0%	DSM-IV-TR	16 weeks, open, parallel; per-protocol analysis	supported by Mazandaran University of Medical Sciences (grant number: 15629)	Vitamin D3 (600000IU/month) +antipsychotics (mean CPZ-equivalent 350mg/day)
Farokhnia M et al., 2013	Acute SCZ outpatients N=42 PANSS baseline > 60 (mean PANSS baseline 113.42 and 114.61 for groups I and II, respectively)	Age: 32.7 F: 52.5%	DSM-IV-TR, SCID	Eight weeks, parallel; intent-to-treat analysis	S.A. has received a grant from Tehran University of Medical Sciences	N-acetylcysteine(2g/day) +risperidone (up to 6mg/day; mean dose 4.2mg/day)
**Sepehrmanesh Z et al.,2017**	Not better-specified phase of illness SCZ outpatients N=84 baseline PANSS >55 (mean baseline PANSS 104 and 87.7 in groups I and II, respectively)	Age: 39 F: 51.8%	DSM-IV-TR, SCID	12 weeks, parallel; per-protocol analysis	funding from the research and technical council of Kashan University of Medical Sciences	N-acetylcysteine(1200mg/day) +antipsychotics
Qiao X et al., 2020	Acute hospitalized aggressive inpatients with SCZ N=67	Age: 34.05 F: 47.7%	ICD-10	Eight weeks, parallel; intent-to-treat analysis	grants from The Three-Year Action Plan for The Construction of Public Health System in Shanghai, the National Natural Science Foundation of China, and Quantitative evaluation strategic research of interventions on relapse of schizophrenia	Omega-3 (EPA540mg/day, DFIA360mg/day) +
Fenton WS et al., 2001	The not better-specified phase of illness SCZ outpatients or schizoaffective disorder and residual symptoms N=87 Mean baseline PANSS 74 and 76 for groups I and II, respectively	Age: 40 F: 39%	DSM-IV	16 weeks, parallel; intent-to-treat analysis	grant from the Stanley Foundation/National Alliance for the Mentally III Research Institute	ethyl EPA(3g/day) +antipsychotics
Pawelczyk T et al., 2016	Acute SCZ Outpatients with the first episode N=71 Mean baseline PANSS 98	Age: 23.2 F: 40.8%	ICD-10, MINI	26 weeks, parallel; intent-to-treat analysis	grant no. N N402 243435 obtained from the Polish Science National Center	Omega-3 (EPA 1.32g/day, DHA 0.88g/day)+antipsychotics (mean CPZ-equivalent 263.16mg/day)
Emsley R et al., 2002	Not better-specified phase of illness SCZ outpatients and persistent symptoms despite six months of antipsychotic treatment N=40 Baseline PANSS >50	Age: 44.9 F: not reported	DSM-IV	12 weeks, parallel; intent-to-treat analysis	grant from the Medical Research Council of South Africa	ethyl EPA(3g/day) +antipsychotics (mean CPZ-equivalent lOllmg/day)
* Emsley R et al., 2014	Clinically stable SCZ outpatients who discontinued antipsychotics after 2–3 years of treatment N=33	Age: 29.7 F: 27.3%	DSM-IV, SCID	Two years, parallel; intent-to-treat analysis	Stanley Medical Research Institute (Grant #09T-1281)	Omega3 (EPA 2g/day, DFIA I g/day) and alpha-lipoic acid (300mg/day). *Note: once randomized, the patient discontinued the antipsychotic treatment.*
Jamilian H et al., 2014	Not better-specified phase of illness SCZ outpatients N=60 Baseline PANSS >60 (mean baseline PANSS 96.13 and 98.26 in groups I and II, respectively)	Age: 31.5 F: 48.35%	DSM-IV-TR, clinical interview	Eight weeks, parallel; intent-to-treat analysis	Deputy of Research of Arak University of Medical Sciences	Omega3 (1 g/day) +antipsychotics
Peet M et al., 2001 **(1)**	Not better-specified phase of illness SCZ outpatients N=45 Baseline PANSS>40	Age: 43.3 F: 33.3%	DSM-IV	12 weeks, parallel; intent-to-treat analysis	Laxdale Limited	EPA(2g/day) +antipsychotics
						DFIA(2g/day) +antipsychotics
Peet M et al., 2001 **(2)**	Not better-specified phase of illness SCZ outpatients N=26	Age: 35.5 F: 40%	DSM-IV	12 weeks, parallel; per-protocol analysis	Funded by Laxdale Limited	EPA(2g/day)+antipsychotics (if necessary)
Peet M et al., 2002	Not better-specified phase of illness SCZ outpatients on persistent ongoing symptoms N=115 PANSS>50	Age: 37 F: 33.91%	DSM-IV	12 weeks, parallel; intent-to-treat analysis	funded by Laxdale Ltd.	ethyl EPA (1–4g/day)+antipsychotics
Tang W et al., 2020	Clinically stable SCZ outpatients N=80 PANSS baseline <60	Age: 28.4 F: 37.5%	DSM-IV, SCID	12 weeks, parallel; per-protocol analysis	supported by the National Key Research and Development Program of China, the National Natural Science Foundation of China, the Shanghai Science and Technology Commission Foundation	Omega3 (EPA720mg/day, DFIA 480mg/day)+olanzapine (mean dose 17.81mg/day)
**Ritener MS et al., 2010**	Clinically stable SCZ outpatients or schizoaffective disorder N=40	Age: 33.3 F: 22.5%	DSM-IV	Eight weeks, parallel; per-protocol analysis	Clinical Trials Grant (#06TGF-911) from the Stanley Medical Research Institute, Bethesda	L-theanine (400mg/day)+antipsychotics (mean CPZ-equivalent 520mg/day)
* Jamilian H et al., 2021	Not better-specified phase of illness SCZ outpatients N=51 Mean baseline PANSS 81.1 and 80.6 in groups I and II, respectively	Age: 45.1 F: not reported	DSM-IV-TR, semi-structured interview not specified	12 weeks, parallel; per-protocol analysis	Arak University of Medical Sciences (AUMS) funds this study	Selenium (200mcg/day) and probiotic (L.acidophilus, B.lactis, B.bifidum, and B.longum, each 2×10^^^9/day
Ghaderi A et al., 2019	The not better-specified phase of illness SCZ outpatients N=60 Mean baseline PANSS 85.4 and 87.5 in groups I and II, respectively	Age: 44 F: 6.7%	DSM-IV-TR, semi-structured interview not specified	12 weeks, parallel; intent-to-treat analysis	The research grant provided by the Research Deputy of Kashan University of Medical Sciences (KAUMS)	VitaminD3 (50000IU/2 weeks) and probiotics (L.acidophilus, B.lactis, B.bifidum, and B.longum, each 2x10^^^9/day)+chlorpromazine (300– 1000mg/day)
Dakhale GN et al., 2005	Acute SCZ outpatients N=40	Age: 38.5 F: not reported	DSM-IV	Eight weeks, parallel; intent-to-treat	Not reported	Vitamin C (500mg/day) +olanzapine (10mg/day) or quetiapine (200mg/day) or ziprasidone(40mg/day)
**Zhang XY et al., 2000**	Not better-specified phase of illness SCZ outpatients N=82	Age: 44.4 F: 42.7%	ICD-10	12 weeks, parallel; intent-to-treat analysis	Not reported	Ginkgo biloba (360mg/day)+haloperidol (12–24 mg/day; mean dose 16.6mg/day)
Doruk A et al., 2008	Not better-specified phase of illness TRS SCZ outpatients N=42	Age: 30.85 F: 33.3%	DSM-IV-TR, SCID	12 weeks, parallel; per-protocol analysis	Not reported	Ginkgo biloba(120mg/day)+clozapine(415mg/day
Chengappa KNR et al., 2018	Acute SCZ outpatients or schizoaffective disorder and psychotic symptoms exacerbation N=68	Age: 46.3 F:48.5%	DSM-IV-TR, MINI	12 weeks, parallel; intent-to-treat analysis	funded by the Stanley Medical Research Institute, Maryland, under grant award 12T-001	Whitania somnífera (1000mg/day) SE+antipsychotics, antidepressants, mood stabilizers, anxiolytics, hypnotics/sedatives (OLA-equivalents 16.39 mg/day for subjects taking atypical antipsychotics)
**Miyaoka T et al., 2014**	Clinically stable SCZ outpatients with treatment-resistant N=117	Age: 46.5 F: 37.6%	DSM-IV-TR, SCID	Four weeks, parallel; intent-to-treat analysis	supported by Grants in Aid for the Ministry of Health, Labor, and Welfare of Japan	Yokukansan (7.5g/day)+ antipsychotics (mean CPZ-equivalent 2037.2mg/day)
Dickerson F et al., 2021	Not better specified phase of illness SCZ outpatients or schizoaffective disorder with stable symptoms (residual psychotic symptoms or at least moderate severity) N=64 Baseline PANSS>60	Age: 44.25 F: 23.5%	DSM-5, SCID	16 weeks, parallel; intent-to-treat analysis	supported by the Stanley Medical Research Institute (15T-001 to FD)	Glucoraphanin 16 mg Tablets (6 Tablets/day) +antipsychotics. 6 Tablets/day yields about 100 μmol of sulforaphane.
Xiao S-F et al., 2011	Not better-specified phase of illness SCZ outpatients and dominant negative symptoms N=80 Mean baseline PANSS 63.9 and 72.1 in groups I and II, respectively	Age: 50.5 F: 51.2%	DSM-IV	Eight weeks, parallel; per-protocol analysis	supported by the grant of Stanley Medical Research Institute (No. 021–005), USA	Sarsasapogenin(200mg/day)+ risperidone (range 2–4mg/day; mean dose 3.33mg/day
Lane K-Y et al., 2006	Not better-specified phase of illness TRS SCZ outpatients N=20 Mean baseline PANSS 78.2 and 77.7 for groups I and II, respectively	Age: 36.1 F: 30%	DSM-IV, SCID	Six weeks, parallel; intent-to-treat analysis	supported by the National Science Council, the National Health Research Institutes, the Development Center for Biotechnology, the National Research Program for Genomic Medicine, the Committee on Chinese Medicine and Pharmacy at the Department of Health, and the China Medical University (Taiwan)	Sarcosine(2g/day)+clozapine (mean dose 306mg/day)
Lane K-Y et al., 2005	Acute SCZ outpatients N=65 The mean baseline PANSS was 86.5 for the sarcosine group, 82.2 for the D-serine group, and 80.7 for the placebo group.	Age: 34 F: 44.4%	DSM-IV, SCID	Six weeks, parallel; per-protocol analysis	supported by grants from the National Science Council (Taipei), the National Health Research Institutes (Taipei), the China Medical University (Taiwan)	Sarcosine (2g/day) +risperidone (mean dose 3.9mg/day)
						D-serine(2g/day) +risperidone(mean dose 4.1mg/day)
Lane H-Y et al., 2010	Not better-specified phase of illness SCZ outpatients N=60 The mean baseline PANSS was 85.3 for the sarcosine group, 88.4 for the D-serine group, and 88.7 for the placebo group	Age: 30.5 F: 40%	DSM-IV, SCID	Six weeks, parallel; per-protocol analysis	funded by the National Science Council (Taiwan), the National Health Research Institutes (Taiwan) and China Medical University (Taiwan)	Sarcosine (2g/day) +risperidone (4.1 mg/day), or quetiapine (400– 600mg/day)
						D-serine(2g/day)+risperidone(4.2mg/day) or olanzapine (20mg/day) or quetiapine (400mg/day)
Tsai GE et al., 2004	Not better-specified phase of illness SCZ outpatients N=38 Mean baseline PANSS 82.6 and 85.2 in groups I and II, respectively	Age: 31.8 F: 39.6%	DSM-IV, SCID	Six weeks, parallel; per-protocol analysis	supported by funding from the National Science Council (Taiwanl, and the National HeaIth Research Institutes (Taiwan)	Sarcosine (2g/day)+antipsychotics (mean CPZ-equivalents 409mg/day)
Stizelecki D et al., 2018	Clinically stable SCZ outpatients with predominantly negative symptoms N=59 Mean baseline PANSS 69.3 and 72.4 for groups I and II, respectively	Age: 38.7 F: 42.4%	DSM-IV-TR and ICD-10, MINI	24 weeks, parallel; intent-to-treat analysis	Polish Ministry of Science and Higher Education (grant N402 268836).	Sarcosine(2g/day)+antipsychotics except for clozapine (defined daily dose 1.94), antidepressants (prescribed daily dose 0.58)
**Lin C-Y et al., 2015**	Clinically stable SCZ outpatients N=63 Mean baseline PANSS was 87.1 for the sarcosine group, 84.9 for the sarcosine+benzoate group, 86.1 for the placebo group	Age: 38.4 F: 38%	DSM-IV	12 weeks, parallel; per-protocol analysis	Hospital Administration Commission, Ministry of Health and Welfare, Taiwan; the Ministry of Science and Technology; Taiwan Ministry of Health and Welfare Clinical Trial and Research Center of Excellence; China Medical University Hospital, Taiwan; Chang-Flua Hospital Program Grant 102–8	Sarcosine(2g/day)+antipsychotics (OLA-equivalents 14.5mg/day)
						Sarcosine(2g/day) and sodium benzoate (1 g/day)+antipsychotics(OLA-equivalents 13.5mg/day)
**Tsai GE et al., 2005**	Not better-specified phase of illness SCZ outpatients N=32 Mean baseline PANSS 80.8 and 82.4 for groups I and II, respectively	Age: 31.4 F: 53.1%	DSM-IV, SCID	Six weeks, parallel; intent-to-treat analysis	Not reported	D-alanine(100mg/kg/day)+antipsychotics (mean CPZ-equivalents 468 mg/day)
Xiao S-H et al., 2012	Not better-specified phase of illness SCZ outpatients with predominant negative symptoms N=101 The mean baseline PANSS was 90.56 and 89.39 in groups I and II, respectively	Age: 48 F:25.7%	DSM-IV	Four weeks, parallel; per-protocol analysis	supported by the Stanley Medical Research Institute (grant no. 021– 005).	Cerebrolysin (30ml i.v/day Monday to Friday)+risperidone (2–4mg/day; mean dos 3.56mg/day)
Ghajar A et al., 2018	Clinically stable SCZ outpatients N=66 The mean baseline PANSS was 47.76 and 48.67 in groups I and II, respectively.	Age: 47.1 F: 10.5%	DSM-5	Eight weeks, parallel; per-protocol analysis	Grant 27749 from Tehran University of Medical Sciences	Citicoline (2500mg/day)+risperidone(mean dose 4.45mg/day)
Turner A et al., 2021	Not better-specified phase of illness SCZ outpatients or schizoaffective disorder N=148 Mean baseline PANSS 73.4 and 70.2 in groups I and II, respectively	Age: 38.8 F: 50%	DSM-5	24 weeks, parallel; intent-to-treat analysis	Stanley Medical Research Institute MD, USA	Mangosteen(1000mg/day)+antipsychotics antidepressants, mood stabilizers, benzodiazepines
Zeinoddini A et al., 2014	Acute SCZ outpatients N=72 Baseline PANSS 105 for both groups	Age: 33.2 F: 68%	DSM-IV-TR, SCID	Eight weeks, parallel; per-protocol analysis	grant 16005 from the Tehran University of Medical Sciences	L-lysine(6g/day) +risperidone (2–6mg/day; mean dose 4.6mg/day)
Tsai GE et al., 1999	Not better-specified phase of illness TRS SCZ outpatients N=20	Age F: 45%	DSM-IV, SCID	Six weeks, parallel; intent-to-treat analysis	Supported by a Young Investigator Award from the National Alliance for Research on Schizophrenia and Depression and a Stanley Foundation Research Award	D-serine+clozapine (mean dose 363mg/day)
D’Souza DC et al., 2013	Clinically stable SCZ outpatients N=104 Mean baseline PANSS 58.14	Age: 37.2 F: 25%	DSM-IV	12 weeks, parallel; intent-to-treat analysis	funding from the Stanley Medical Research Institute, the Donaghue Foundation, a VA Career Award, the VA Schizophrenia Center, and the VA Cooperative Studies Program	D-serine (30mg/kg/day)+CRT+antipsychotics D-serine (30mg/kg/day)+controlCRT+antipsychotici
Heresco-Levy U et al., 2005	Clinically stable TRS SCZ outpatients N=39 Mean baseline PANSS 100.7 and 103.7 in groups I and II, respectively	Age: 44.8 F: 35.9%	DSM-IV, semi-structured psychiatric interview	Six weeks, crossover; intent-to-treat analysis	supported by a research award from the Stanley Medical and Research Institute, Bethesda	D-serine (30mg/kg/day)+olanzapine or risperidone
Evins AE et al., 2000	Clinically stable SCZ outpatients N=27 Mean baseline PANSS 70 and 75 in groups I and II, respectively	Age: 39 F: 22.2%	DSM-IV	Eight weeks, parallel; intent-to-treat analysis	Supported by a National Alliance for Research on Schizophrenia and Depression Young Investigator Award	Glycine (60g/day)+clozapine
Serrita J et al., 2019	Clinically stable SCZ outpatients or schizoaffective disorder and AUD N=20 Mean baseline PANSS 65.4 and 57.3 for groups I and II, respectively	Age: 48.8 F: 0%	DSM-IV, SCID	12 weeks, parallel; intent-to-treat analysis	Stanley Foundation (grant # 02T-241) and Veterans Affairs VISN I Mental Illness Research Education and Clinical Center (MIRECC)	Glycine (0.8/kg/day)+antipsychotics excep for clozapine
Buchanan RW et al., 2007	Clinically stable SCZ outpatients or schizoaffective disorder with persistent negative symptoms N=157	Age: 43.4 F: not reported	DSM-IV, SCID	16 weeks, parallel; intent-to-treat analysis	Supported by the Treatment of Negative Symptoms and Cognitive Impairments grants; Advanced Center for Intervention and Services Research grant; and VA Capitol Network MIRECC	Glycine (60g/day)+ antipsychotics, mood stabilizers, antidepressants, anxiolytics, anticholinergic drugs D-cycloserine (50mg/day)+antipsychotics, mood stabilizers, antidepressants, anxiolytics, anticholinergic drugs

**Abbreviations:** AIMS=Abnormal Involuntary Movements Scale; BARS=Barnes Akathisia Rating Scale; BDNF=Brain Derived Neurotrophic Factor; BPRS=Brief Psychiatric Rating Scale; CANTAB=Cambridge Automated Neuropsychological Test Battery; CDSS=Calgary Depression Scale for Schizophrenia; CGI=Cllnlcal Global Impression; DHA=docosahexaenoic acid; EPA=eicosatetraenoic acid; ESRS=Extrapyramidal Symptoms Rating Scale; GAF=Global Assessment of Functioning; GAS=Global Assessment Scale; HDRS=Flamilton Depression rating Scale; LIFE-RIFT=Range of Impaired Functioning Tool; MATRICS=Measurement and Treatment Research to Improve Cognition in Schizophrenia; MOAS= Modified Overt Aggression Scale; MoCA= Montreal Cognitive Assessment; PSS=Perceived Stress Scale; Q-LES-Q18=Quality of Life Enjoyment and Satisfaction Questionnaire; QLS=Quality of Life Scale; QOL=Quality Of Life; RBANS=RepeaTable Battery for Assessment of Neuropsychological Status; SANS=Scale for Assessment of Negative Symptoms; SAPS=Scale for Assessment of Positive Symptoms; SAS=Simpson Angus Scale; SCZ=schizophrenia; SOFAS=Social and Occupational Functioning Assessment Scale; TRS=Treatment Resistant Schizophrenia; WAIS=Wechsler Adult Intelligence Scale.

* Study entering the SR portion only.
